# Fetal Heart Rate at 12 Weeks' Gestation and the Risk of Preterm Birth: A Prospective Validation Study

**DOI:** 10.1002/jum.70252

**Published:** 2026-04-13

**Authors:** José Morales‐Roselló, Alicia Soriano‐Payá, Blanca Novillo‐Del Álamo, Alicia Martínez‐Varea

**Affiliations:** ^1^ Servicio de Obstetricia y Ginecología Hospital Universitario y Politécnico La Valencia Spain; ^2^ Departamento de Pediatría Obstetricia y Ginecología, Universidad de Valencia Valencia Spain; ^3^ Servicio de Obstetricia y Ginecología Hospital Universitario Francisco de Borja Valencia Spain; ^4^ Departamento de Medicina y Cirugía Facultad de Ciencias de la Salud, Universidad Cardenal Herrera‐CEU Valencia Spain

**Keywords:** fetal heart rate, first trimester, free‐βhCG, preterm birth, spontaneous preterm birth

## Abstract

**Objectives:**

To prospectively validate the association between fetal heart rate (FHR) at the 12‐week scan and the risk of preterm birth (PTB), including spontaneous preterm birth (sPTB).

**Methods:**

This prospective cohort study included 1276 singleton pregnancies undergoing routine first‐trimester screening at 11–13 + 6 weeks' gestation and followed until delivery. Associations between FHR and PTB outcomes were assessed using univariable and multivariable logistic regression analyses and receiver operating characteristic (ROC) curves, incorporating clinical, sonographic, and biochemical first‐trimester parameters.

**Results:**

Compared with term births, pregnancies resulting in PTB (*n* = 34) and sPTB (*n* = 23) showed significantly higher FHR at the 12‐week scan (163.6 versus 160.1 bpm, *p* = .007; and 164.1 versus 160.1 bpm, *p* = .014, respectively). In univariable analyses, FHR was the only parameter consistently associated with PTB, PTB <34 weeks, sPTB, and sPTB <34 weeks (odds ratios per bpm increase ranging from 1.08 to 1.12). Free β‐human chorionic gonadotropin (free‐βhCG) was associated with PTB <34 weeks and showed borderline significance for other PTB outcomes. In multivariable models, FHR showed the highest predictive performance for sPTB <34 weeks (AUC 0.73; detection rate 25% at a 10% false‐positive rate), which improved when combined with free‐βhCG (AUC 0.81; detection rate 43% at a 10% false‐positive rate).

**Conclusion:**

Elevated FHR at the 12‐week scan is associated with sPTB, particularly before 34 weeks' gestation. FHR, especially when combined with free‐βhCG, may contribute to early first‐trimester screening strategies for sPTB.

**Abbreviations**
AIC: Akaike information criterion
AUC: area under the curve
CRL: crown–rump length
DR: detection rate
FHR: fetal heart rate
FPR: false‐positive rate
GA: gestational age
IQR: interquartile range
NT: nuchal translucency
PAPP‐A: pregnancy‐associated plasma protein‐A
PTB: preterm birth
ROC: receiver operating characteristic
sPTB: spontaneous preterm birth

Spontaneous preterm birth (sPTB) is a heterogeneous inflammatory syndrome that results in delivery at an early gestational age (GA) and remains a leading cause of neonatal morbidity and mortality worldwide.^1^ Although sPTB can be triggered by multiple mechanisms, inflammation, often related to local or distant infection, is considered a central pathway initiating the cascade leading to preterm labor.^2^, ^3^, ^4^, ^5^, ^6^, ^7^, ^8^, ^9^ Current screening strategies for sPTB rely mainly on mid‐pregnancy assessment, particularly transvaginal measurement of cervical length at approximately 20 weeks' gestation.^10^, ^11^ However, at this stage, the biological processes leading to preterm labor may already be established, potentially limiting the effectiveness of preventive interventions. This limitation underscores the need to identify reliable first‐trimester markers that allow for earlier risk stratification and timely primary prevention.

In a recent retrospective study conducted in a tertiary referral population, we reported an association between first‐trimester fetal heart rate (FHR) and preterm birth (PTB) across different GA thresholds.^12^ Given the simplicity, reproducibility, and universal availability of FHR measurement during routine first‐trimester ultrasound, validation of this finding in a prospective and independent population is essential.

The aim of the present study was therefore to prospectively validate the previous association between FHR at the 12‐week scan and the risk of PTB and sPTB in a regional hospital setting, incorporating not only clinical and sonographic parameters but also biochemical markers routinely used for first‐trimester aneuploidy screening.

## Study Design and Population

This prospective cohort study included 1276 singleton pregnancies undergoing routine first‐trimester evaluation at Hospital Universitario Francisco de Borja (Gandía, Spain). All pregnancies were examined between 11 and 13 + 6 weeks' gestation and followed until delivery.

The first‐trimester assessment included measurement of crown–rump length (CRL) for pregnancy dating, nuchal translucency (NT), and FHR, as well as biochemical analysis of pregnancy‐associated plasma protein‐A (PAPP‐A) and free β‐human chorionic gonadotropin (free‐βhCG), obtained approximately 2 weeks earlier as part of the combined screening for aneuploidy. Maternal characteristics recorded included age, pre‐pregnancy weight and height, parity, GA at examination, and fetal sex.

Ultrasound examinations were performed using a General Electric Voluson® E8 system with 2–8 MHz transabdominal convex probes.

### Exclusion Criteria

**Figure 1:**
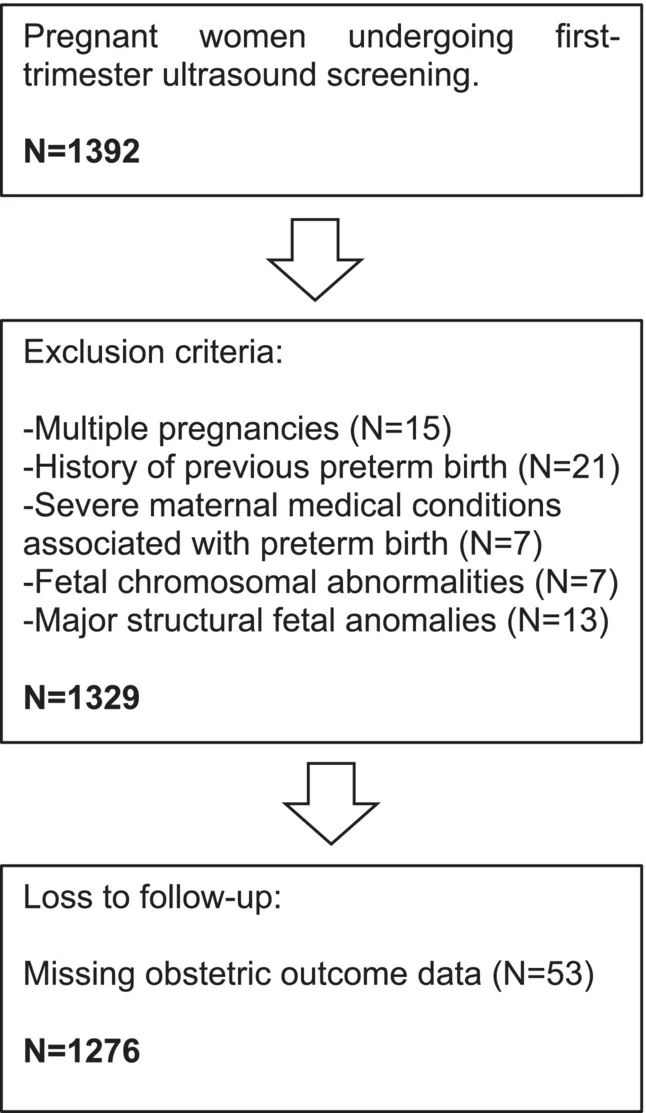
Flow chart illustrating the inclusion and exclusion of participants in the study population.

Multiple pregnancies, pregnancies with a history of previous PTB, maternal chronic conditions predisposing to PTB, and pregnancies complicated by major fetal structural anomalies or chromosomal abnormalities were excluded (Figure 1).

### Follow‐Up and Outcome Assessment

Pregnancies were managed according to local hospital protocols^10^ and followed until delivery. Outcome data collected included GA at birth, birthweight, birthweight centile, mode of labor onset (spontaneous, induction, or cesarean section), mode of delivery, Apgar score, and umbilical arterial pH.

### Definitions of Outcomes

Preterm birth (PTB) was defined as delivery before 37 weeks' gestation, and PTB <34 weeks as delivery before 34 weeks, irrespective of the mode of onset or medical indication.

sPTB and sPTB <34 weeks were strictly defined as deliveries occurring spontaneously before 37 and 34 weeks' gestation, respectively, excluding all cases of medically indicated induction or cesarean delivery for maternal or fetal complications.

### Statistical Analysis

Continuous variables are presented as median and interquartile range (IQR), and categorical variables as number and percentage. Comparisons between groups were performed using the Mann–Whitney *U* test or Kruskal–Wallis test for continuous variables and the *χ* ^2^ test for categorical variables.

Univariable logistic regression analyses were conducted to assess the association of each first‐trimester parameter with PTB, sPTB, PTB <34 weeks, and sPTB <34 weeks. Variables showing statistical significance or borderline significance were entered into multivariable logistic regression models. Model performance was assessed using receiver operating characteristic (ROC) curves, area under the curve (AUC), detection rate (DR), false‐positive rate (FPR), and Akaike information criterion (AIC). A difference of ≥2 units in AIC was considered significant.

Statistical analyses were performed using StatPlus® (version 7) and GraphPad Prism® (version 9). Statistical significance was set at *p* < .05.

### Ethical Approval

The study was approved by the ethics committees of Hospital Universitario Francisco de Borja (registry number 8/2021) and Hospital Universitario y Politécnico La Fe (registry number 2022‐555‐1).

## Results

### Study Population

Baseline characteristics of the study population are summarized in Table 1. The mean maternal age was 31.2 years, mean pre‐pregnancy weight 71.1 kg, mean height 163 cm, mean parity 0.72, and mean GA at delivery was 39.6 weeks. Overall, 16.9% of women reported smoking during pregnancy, 6.3% conceived by assisted reproductive techniques, 49.0% were nulliparous, and 15.4% had hypothyroidism diagnosed during pregnancy.

**Table 1:** Description of the Study Population (*N* = 1276) and the Population Presenting Any Preterm (*N* = 34), Spontaneous Preterm (*N* = 23), and Term Births (*N* = 1242)

Parameter	1. All Pregnancies (*N* = 1276)	2. Any Preterm Birth (*N* = 34)	3. Spontaneous Preterm Birth (*N* = 23)	4. Term Birth (*N* = 1242)	2 vs 4	3 vs 4
	Mean (SD); median (1st, 3rd quartile)	Mean (SD); median (1st, 3rd quartile)	Mean (SD); median (1st, 3rd quartile)	Mean (SD); median (1st, 3rd quartile)	*p*‐Value^a^	*p*‐Value^a^
Maternal age (year)	31.2 (5.96); 32.0 (27.0, 35.0)	31.06 (5.4); 30.5 (27.75, 36.25)	31.1 (5.2); 30.0 (28.0,36.0)	31.16 (5.9); 32 (27, 35)	.7701	.7754
Maternal pre‐pregnancy weight (kg)	71.1 (14.9); 69.5 (60, 79)	75.12 (20.5); 70.5 (58.7, 90.2)	70.4 (16.1); 67.2 (58.5, 87.3)	71 (14.7); 69.5 (60.1, 79)	.4704	.6555
Maternal height (cm)	163.0 (5.9); 163 (159, 167)	163.3 (6.4); 163 (157, 168)	163.2 (6.4); 163.0 (157.0, 168.0)	163.0 (5.9); 163 (159, 167)	.7468	.9726
Parity	0.72 (0.87); 1 (0, 1)	0.62 (0.85); 0 (0, 1)	0.69 (0.92); 0 (0, 1)	0.73 (0.87); 1 (0, 1)	.3874	.7311
Gestational age at ultrasound scan (week)	12.44 (0.44); 12.28 (12.0, 12.71)	12.4 (0.46); 12.28 (12.0, 12.71)	12.34 (0.39); 12.28 (12.0, 12.57)	12.44 (0.44); 12.28 (12, 12.71)	.6726	.4604
PAPP‐A (mU/mL)	3.575 (2.187); 3.11 (1.96, 4.71)	3.39 (2.592); 2.70 (1.57, 4.178)	3.89 (2.93); 3.04 (1.73, 5.73)	3.58 (2.18); 3.12 (1.98, 4.72)	.2349	.9267
Free‐βhCG (mU/mL)	44.9 (267.9); 29.7 (19.32, 46.32)	46.6 (41.27); 34 (20.6, 58.86)	49.2 (47.9); 33.2 (21.4, 57.3)	37.0 (27.95); 29.56 (19.25, 45.89)	.1316	.2253
Leukocytes count (μL)	8357 (1991); 8200 (7000, 9500)	8434 (1545); 8450 (7325, 9275)	8473 (1673); 8450 (7200, 9350)	8355 (2001); 8200 (7000, 9500)	.5851	.6275
Neutrophils count (μL)	5311 (1603); 5200 (4200, 6200)	5316 (1019); 5250 (4550, 5775)	5286 (1080): 5200 (4475, 5700)	5311 (1616); 5200 (4200, 6300)	.7393	.8990
Lymphocytes count (μL)	2304 (1190); 2200 (1800, 2600)	2334 (661); 2250 (1800, 2600)	2377 (707); 2350 (1775, 2800)	2303 (1201); 2200 (1800, 2600)	.7096	.5452
Nuchal translucency (mm)	1.55 (0.39); 1.50 (1.30, 1.75)	1.61 (0.32); 1.58 (1.39, 1.74)	1.68 (0.34); 1.70 (1.40, 1.90)	1.55 (0.39); 1.50 (1.30, 1.76)	.2568	.0788
Crown rump length (mm)	63.3 (16.0); 62.5 (58.1, 67.3)	62.5 (6.6) 62.6 (56.8, 67.0)	62.2 (6.28); 62.5 (56.8, 66.1)	63.35 (16.23); 62.5 (58.1, 67.3)	.6377	.5389
Fetal heart rate (bpm)	160.2 (7.07); 160 (156, 164)	163.6 (1.12); 163.5 (158.8, 167.3)	164.1 (6.8); 164 (159, 168)	160.1 (7.06); 160.0 (155.0, 164.0)	.0072	.0144
Gestational age at delivery (week)	39.6 (1.6); 39.8 (38.8, 40.6)	33.74 (3.5); 35.07 (33.03, 36.04)	33.56 (3.7); 35.0 (32.7, 36.0)	39.76 (1.12); 39.85 (39, 40.57)	<.0001	<.0001
Birth weight (g)	3337 (502.8); 3340 (3046, 3660)	2246 (696.7); 2397 (1879, 2688)	2236 (737); 2394 (1860, 2670)	3367 (461.8); 3350 (3065, 3670)	<.0001	<.0001
Birth weight centile	51.1 (30.8) 51 (24, 79)	46.91 (31.25); 44.5 (17.25, 78.25)	48.3 (31.5); 46.0 (25.0, 82.0)	51.24 (30.8); 51.0 (24.0, 79.0)	.4124	.6633

	*N*/total (%)	*N*/total (%)	*N*/total (%)	*N*/total (%)	*p*‐Value^b^	*p*‐Value^b^
Nulliparity	626/1276 (49.0)	20/34 (58.8)	13/23 (56.5)	606/1242 (48.8)	.2977	.4625
Fetal male sex	678/1276 (53.1)	22/34 (64.7)	15/23 (65.2)	688/1242 (55.4)	.2998	.3475
Smoking	215/1274 (16.9)	4/34 (11.8)	3/23 (13.0)	211/1242 (17)	.6408	.6170
Assisted reproduction^c^	81/1276 (6.3)	2/34 (5.9)	1/23 (4.3)	79/1242 (6.4)	1.0000	.6943
Hypothyroidism^d^	197/1276 (15.4)	7/34 (20.6)	7/23 (30.4)	190/1242 (15.3)	.4671	.0473
Rh‐	176/1276 (13.8)	4/34 (11.8)	4/23 (17.4)	172/1242 (13.8)	1.0000	.6267
Blood antigen A	580/1276 (45.4)	16/34 (47.0)	13/23 (56.5)	564/1242 (45.4)	.8631	.2891
Blood antigen B	225/1276 (17.6)	8/34 (23.5)	5/23 (21.7)	217/1242 (17.5)	.3618	.5940
Type of labor onset						
Cesarean section	109 /1276 (8.5)	2/34 (5.9)	0/23 (0%)	107/1242 (8.6)	.7630	.1412
Induction of labor	442/1276 (34.6)	9/34 (26.5)	0/23 (0%)	702/1242 (56.5)	.0007	<.0001
Spontaneous onset of labor	725/1276 (56.8)	23/34 (67.6)	23/23 (100%)	433/1242 (34.9)	.0002	<.0001
Mode of birth						
Cesarean section	370/1276 (29)	8/34 (23.5)	3/23 (13.0)	362/1242 (29.1)	.5683	.0912
Assisted vaginal delivery	184/1276 (14.4)	4/34 (11.8)	4/23 (17.4)	180/1242 (14.5)	.8077	.6960
Spontaneous vaginal delivery	722/1276 (56.6)	22/34 (64.7)	16/23 (69.6)	700/1242 (56.4)	.3832	.2055
Apgar at 5 minutes <7	5/1260 (0.40)	4/34 (11.8)	3/23 (13)	1/1226 (0.1)	<.0001	<.0001
Arterial pH <7.10	6/1036 (0.58)	2/25 (5.9)	2/17 (11.8)	4/1011 (0.4)	.0079	<.0001

^a^ Mann–Whitney *U* test.

^b^ Chi‐square test, SD: standard deviation.

^c^ Any assisted reproduction treatment.

^d^ Pregestational or gestational hypothyroidism, PAPP‐A, pregnancy‐associated plasma protein‐A; free‐βhCG, free β‐human chorionic gonadotropin; bpm, beats per minute.

Most pregnancies had spontaneous onset of labor and vaginal delivery. Cesarean section was performed in 29.8% of cases, of which 8.5% were elective. Adverse neonatal outcomes were infrequent, with umbilical arterial pH <7.0 or Apgar score <7 at 5 minutes occurring in fewer than 1% of cases.

Among the 1276 pregnancies, 34 (2.7%) resulted in PTB and 23 (1.8%) in sPTB.

### Fetal Heart Rate and Preterm Birth

**Figure 2:**
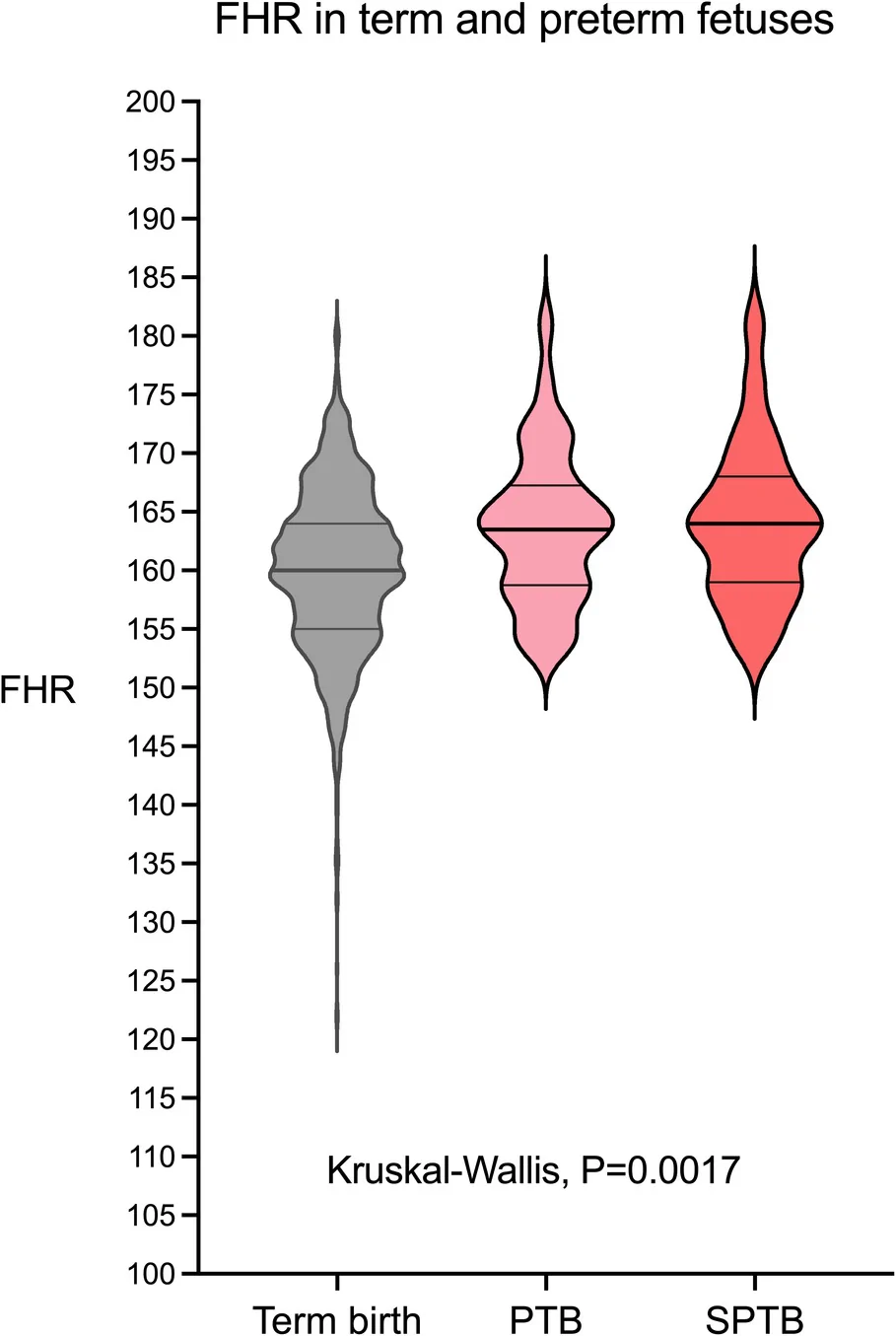
Violin plots showing the fetal heart rate of fetuses presenting term, preterm, and spontaneous preterm births.

Pregnancies ending in PTB and sPTB showed significantly higher FHR at the 12‐week scan compared with term pregnancies (163.6 versus 160.1 bpm, *p* = .007, and 164.1 versus 160.1 bpm, *p* = .014, respectively; Table 1, Figure 2). This difference remained evident when restricting the analysis to births before 34 weeks.

A higher prevalence of maternal hypothyroidism was observed in the sPTB group compared with term pregnancies (30.4% versus 15.3%, *p* = .047), whereas no significant differences were found for other maternal or sonographic variables.

### Univariable Analysis

Results of the univariable logistic regression analyses are shown in Tables 2 and 3. Fetal heart rate was the only parameter consistently associated with all PTB outcomes, including PTB, PTB <34 weeks, sPTB, and sPTB <34 weeks, with odds ratios per bpm increase ranging from 1.08 to 1.12.

**Table 2:** Univariable Logistic Regression Analysis for the Prediction of Any Cause of Preterm Birth Before 37 and 34 Weeks Using the Parameters Available at the 12‐Week Screening Scan

Parameter	Intercept	Estimate	Stand Error	OR (95% CI)	*p*‐Value
Preterm birth <37 weeks					
Maternal data					
Maternal age	−3.5072	−0.0029	0.0291	0.9971 (0.9417, 1.0557)	.9201
Maternal pre‐pregnancy weight	−4.8589	0.0169	0.0119	1.0171 (0.9936, 1.0411)	.1545
Maternal height	−4.8754	0.0077	0.0329	1.0077 (0.9447, 1.0749)	.8152
Parity	−3.4920	−0.1579	0.2162	0.8539 (0.5589, 1.3045)	.4649
Smoking	−3.5351	−0.4304	0.5376	0.6502 (0.2267, 1.8651)	.4233
Assisted reproduction^a^	−3.5930	−0.0833	0.7381	0.9200 (0.2165, 3.9093)	.9102
Hypothyroidism^b^	−3.7003	−12.0414	917.4394	0.0000 (0,0000, N/A)	.9895
Ultrasound data					
Fetal heart rate	−16.4128	0.0791	0.0267	1.0824 (1.0271, 1.1406)	.0031
Nuchal translucency	−4.1844	0.3706	0.4135	1.4485 (0.6440, 3.2579)	.3702
Crown rump length	−3.0317	−0.0090	0,0252	0,9910 (0,9431, 1,0413)	.7210
Fetal sex male	−3.8918	0.4998	0,3633	1,6483 (0.8086, 3.3599)	.1690
Blood test data					
PAPP‐A	−3.4597	−0.0402	0.0867	0.9606 (0.8105, 1.1385)	.6429
Free β‐hCG	−3,9291	0.0080	0.0043	1.0081 (0.9996, 1.0166)	.0631
Leukocytes count	−3.8087	0.0199	0.0890	1.0201 (0,8568, 1.2145)	.8231
Neutrophils count	−3.6516	0.0019	0,1116	1.0019 (0.8049, 1.2469)	.9867
Lymphocytes count	−3.6851	0.0187	0.1268	1.0189 (0.7946, 1.3065)	.8829
Rh‐	−3.5733	−0.1879	0.5386	0.8287 (0.2884, 2.3815)	.7272
Blood antigen A	−3.6288	0.0663	0.3483	1.0685 (0.5399, 2.1148)	.8490
Blood antigen B	−3.6743	0.3739	0.4111	1.4534 (0.6492, 3.2536)	.3631
Preterm birth <34 weeks					
Maternal data					
Maternal age	−4.0845	−0.0186	0.0482	0.9816 (0.8932, 1.0788)	.6998
Maternal pre‐pregnancy weight	−3.3222	−0.0189	0.0237	0.9813 (0,9368, 1.0279)	.4241
Maternal height	−5,5858	0,0059	0.0537	1.0059 (0.9054, 1.1175)	.9131
Parity	−4.8404	0.2245	0.2959	1.2517 (0.7009, 2.2354)	.4479
Smoking	−4.5567	−0.8092	1.0472	0.4452 (0.0572, 3.4667)	.4396
Assisted reproduction^a^	−4.6788	0.2967	1.0508	1,3454 (0,1715, 10,5525)	.7776
Hypothyroidism^b^	−4,7782	0.6089	0.6712	1.8385 (0.4933, 6.8519)	.3643
Ultrasound data					
Fetal heart rate	−19.2057	0.0897	0.0445	1.0938 (1.0024, 1.1936)	.0440
Nuchal translucency	−5.3366	0.4279	0.6799	1.5339 (0.4046, 5.8157)	.5292
Crown rump length	−4.7811	0.0019	0.0118	1.0019 (0.9791, 1.0253)	.8685
Fetal sex male	−4.7791	0.2192	0.5882	1.2451 (0.3931, 3.9441)	.7094
Blood test data					
PAPP‐A	−4.9513	0.0708	0.1241	1.0734 (0,8416, 1.3690)	.5682
Free β‐hCG	−5.1877	0.0114	0.0056	1.0115 (1.0004, 1.0227)	.0427
Leukocytes count	−5.9009	0.1359	0.1401	1.1457 (0.8705, 1.5078)	.3318
Neutrophils count	−5.3442	0.1131	0.1769	1.1198 (0.7915, 1.5841)	.5227
Lymphocytes count	−4.8665	0.0591	0.1379	1.0609 (0,8096, 1.3902)	.6680
Rh‐	−4.4659	−0.2254	0.7789	0.7982 (0.1734, 3.6736)	.7722
Blood antigen A	−4.7449	0.1841	0.5801	1.2021 (0.3856, 3.7474)	.7510
Blood antigen B	−4.8704	0.8585	0.6168	2.3597 (0.7044, 7.9053)	.1639

OR, odds ratio; Stand Error, standard error; CI, confidence interval.

^a^ Any assisted reproduction treatment.

^b^ Pregestational or gestational hypothyroidism, PAPP‐A, pregnancy associated plasma protein‐A; free β‐hCG, free beta human chorionic gonadotropin.

**Table 3:** Univariable Logistic Regression Analysis for the Prediction of Spontaneous Preterm Birth Before 37 and 34 Weeks Using the Parameters Available at the 12‐Week Screening Scan

Parameter	Intercept	Estimate	Standard E	OR (95% CI)	*p*‐Value
Spontaneous preterm birth <37 weeks					
Maternal data					
Maternal age	−3.9327	−0.0021	0.0353	0.9979 (0.9312, 1.0694)	.9527
Maternal pre‐pregnancy weight	−3.7514	−0.0032	0.0158	0.9968 (0.9663, 1.0282)	.8381
Maternal height	−4.9935	0.0062	0.0391	1.0062 (0.9319, 1.0864)	.8737
Parity	−3.9686	−0.0410	0.2463	0.9598 (0.5922, 1.5554)	.8677
Smoking	−3.9502	−0.3077	0.6237	0.7351 (0.2165, 2.4962)	.6218
Assisted reproduction^a^	−3.9763	−0.4057	1.0289	0.6665 (0.0887, 5.0081)	.6933
Hypothyroidism^b^	−4.1963	0.8951	0.4599	2.4477 (0.9936, 6.0295)	.0516
Ultrasound data					
Fetal heart rate	−18.4048	0.0889	0.0324	1.0929 (1.0256, 1.1647)	.0062
Nuchal translucency	−5.1729	0.7285	0.4671	2.0720 (0.8294, 5.1763)	.1188
Crown rump length	−2.9943	−0.0160	0.0312	0,9841 (0,9257, 1,0462)	.6078
Fetal sex male	−4.3041	0.5184	0.4414	1,6793 (0.7069, 3.9892)	.2403
Blood test data					
PAPP‐A	−4.2116	0.0614	0.0898	1.0633 (0.8917, 1.2678)	.4942
Free β‐hCG	−4.3709	0.0092	0.0048	1.0093 (0.9999, 1.0187)	.0518
Leukocytes count	−4.2712	0.0293	0.1063	1.0297 (0.8361, 1.2683)	.7827
Neutrophils count	−3.9726	−0.0098	0.1349	0.9902 (0.7602, 1.2899)	.9421
Lymphocytes count	−4.1101	0.0366	0.1268	1.0373 (0.8091, 1.3299)	.7725
Rh‐	−3.7612	−0.2800	0.5562	0.7558 (0.2541, 2.483)	.6147
Blood antigen A	−4.2283	0.4529	0.4244	1.5728 (0.6845, 3.6138)	.2859
Blood antigen B	−4.0498	0.2657	0.5109	1.3043 (0.4791, 3.5506)	.6031
Spontaneous preterm birth < 34 weeks					
Maternal data					
Maternal age	−4.9251	−0.0045	0.0594	0.9955 (0.8861, 1.1184)	.9393
Maternal pre‐pregnancy weight	−3.8133	−0.0149	0.0259	0.9851 (0.9363, 1.0364)	.5627
Maternal height	−11.2947	0.0393	0.0594	1.0401 (0.9258, 1.1686)	.5079
Parity	−5.3283	0.3072	0.3469	1.3597 (0.6889, 2.6834)	.3757
Smoking	−4.8781	−15.1961	945,5379	0 (0, N/A)	.9872
Assisted reproduction^a^	−5.1341	0.7521	1.0753	2.1214 (0,2578, 17,4548)	.4843
Hypothyroidism^b^	−5.3697	1.2005	0.7344	3.3216 (0.7874, 14.0130)	.1021
Ultrasound data					
Fetal heart rate	−23.3101	0.1121	0.0548	1.1187 (1.0047, 1.2455)	.0407
Nuchal translucency	−6.7011	0.9968	0.7392	2.7097 (0.6363, 11.5389)	.1775
Crown rump length	−5.1698	0.0016	0.0154	1.0016 (0.9719, 1.0323)	.9153
Fetal sex male	−5.2933	0.3939	0.7325	1.4829 (0.3529, 6.2314)	.5907
Blood test data					
PAPP‐A	−5.6367	0.1266	0.1407	1.1350 (0,8613, 1.4957)	.3685
Free β‐hCG	−5,6372	0.0113	0.0069	1.0113 (0.9978, 1.0251)	.1014
Leukocytes count	−5.8794	0.0972	0.1680	1.1021 (0.7928, 1.5321)	.5628
Neutrophils count	−5.3320	0.0529	0.2149	1.0543 (0.6919, 1.6065)	.8055
Lymphocytes count	−5.1651	0.0500	0.1775	1.0513 (0,7423, 1.4889)	.7782
Rh‐	−4.4659	−0.7399	0.8206	0.4771 (0.0955, 2.3830)	.3672
Blood antigen A	−5.4424	0.6975	0,7325	2.0087 (0.4779, 8.4413)	.3409
Blood antigen B	−5.1600	0.4459	0.8198	1.5620 (0.3132, 7.7899)	.5864

OR, odds ratio; Stand Error, standard error; CI, confidence interval.

^a^ Any assisted reproduction treatment.

^b^ Pregestational or gestational hypothyroidism, PAPP‐A, pregnancy associated plasma protein‐A; free β‐hCG, free beta human chorionic gonadotropin.

Free‐βhCG was significantly associated with PTB <34 weeks and showed borderline significance for PTB and sPTB. Maternal hypothyroidism showed borderline significance only for sPTB and was not associated with other outcomes.

### Multivariable Analysis and Model Performance

**Figure 3:**
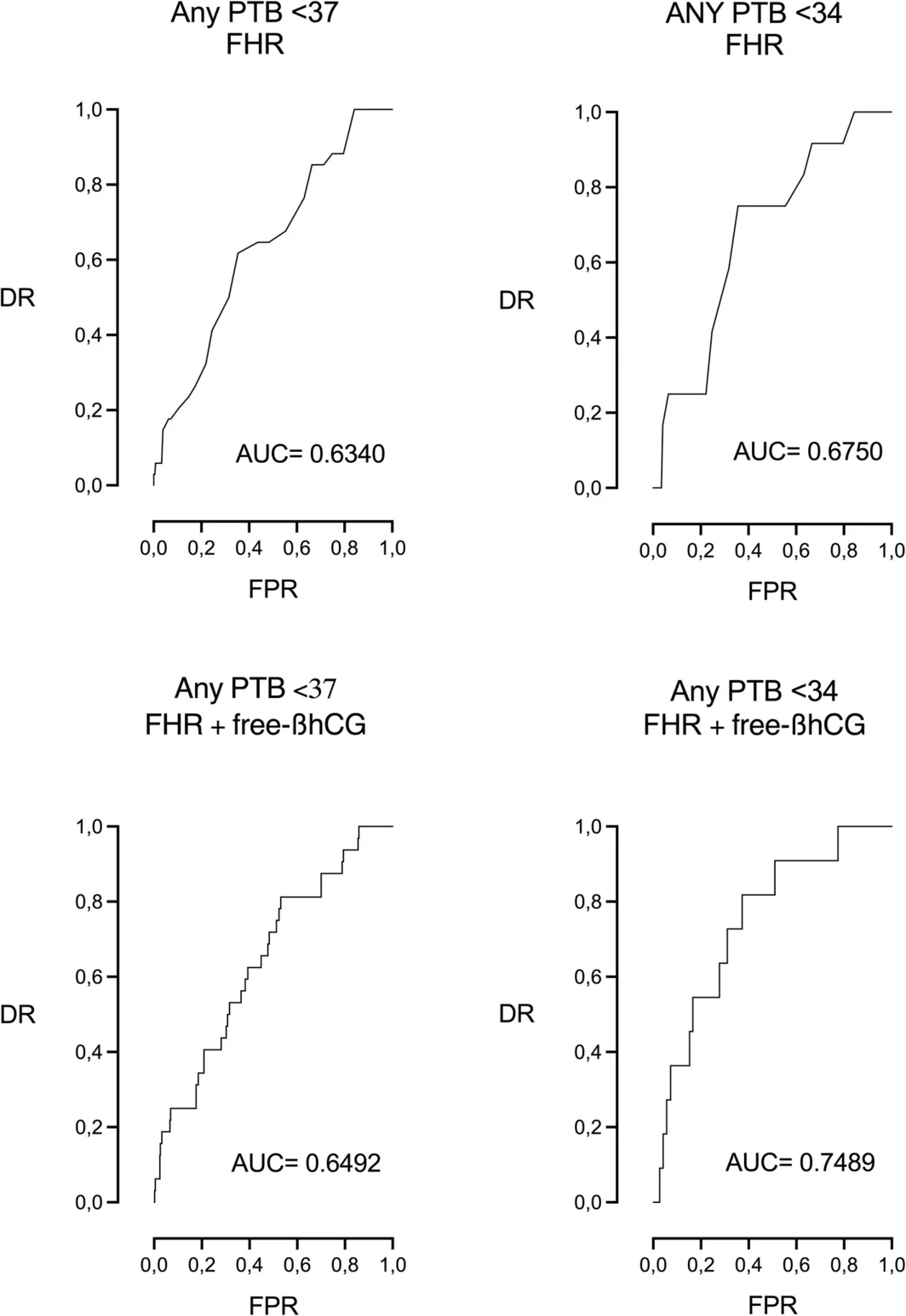
ROC curves of several models for the prediction of any preterm birth (PTB). In the upper part, the fetal heart rate (FHR) alone before 37 and 34 weeks, and in the lower part, the FHR combined with the free‐bhCG before 37 and 34 weeks.

**Figure 4:**
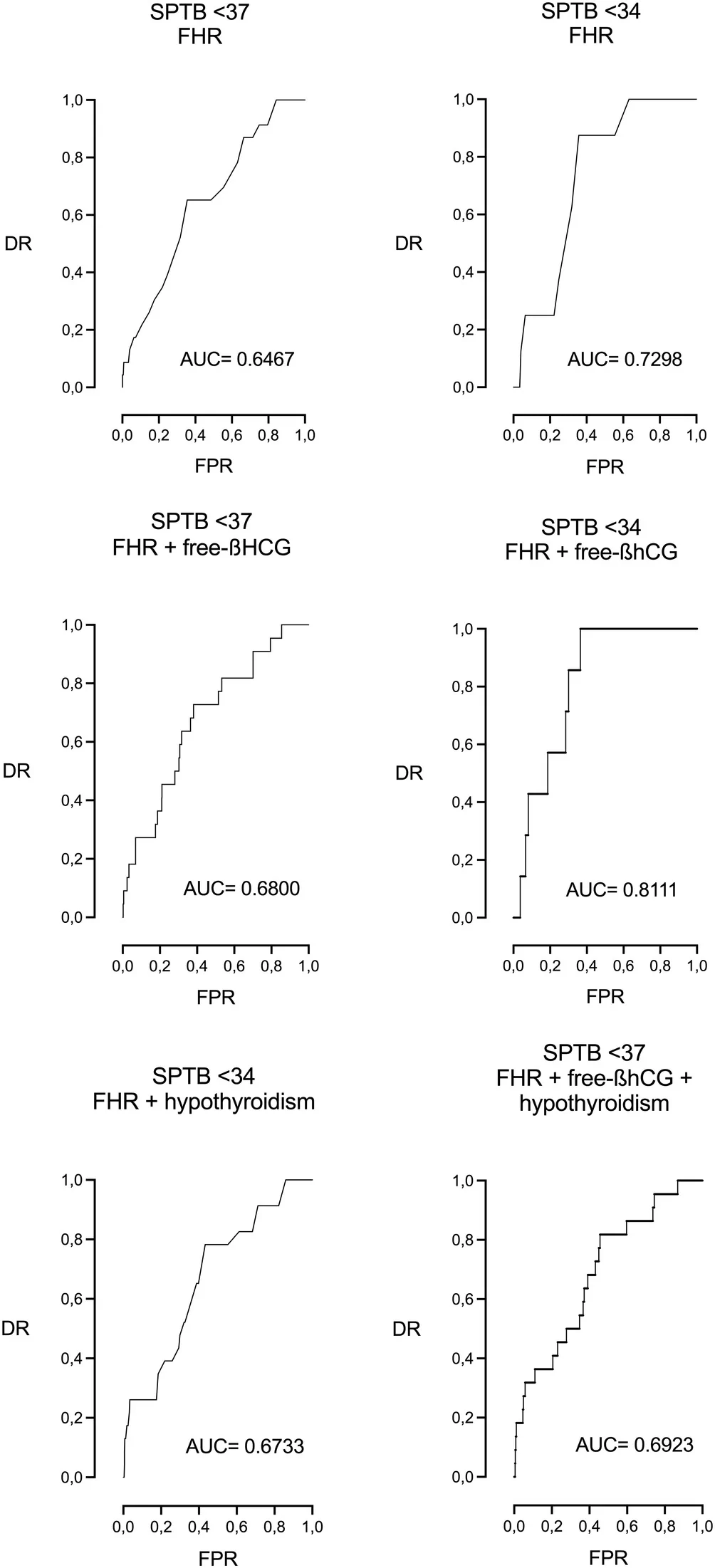
ROC curves of several models for the prediction of spontaneous preterm birth (sPTB). In the upper part, the fetal heart rate (FHR) alone before 37 and 34 weeks, in the middle part, the FHR combined with the free‐bhCG before 37 and 34 weeks, and in the lower part, the FHR combined with the maternal thyroid status (hypothyroidism) <34 weeks, and the FHR combined with the free‐bhCG and the maternal thyroid status (hypothyroidism) <37 weeks.

Multivariable models including variables with significant or borderline significance in univariable analyses are presented in Table 4 and Figures 3 and 4.

**Table 4:** Multivariable Logistic Regression Analysis Using Those Parameters that Were Statistically Significant or Borderline Significant in the Univariable Analysis

	Estimate	Standard E	OR (95% CI)	*p*‐Value
Any cause of preterm birth				
Preterm birth <37 weeks				
Fetal heart rate				
Fetal heart rate	0.0791	0.0267	1.0824 (1.0271, 1.1406)	.0031
Intercept	−16.4128			
AUC = 0.6340, (95% CI = 0.5437, 0.7242), AIC = 308.5, DR = 16% for a FPR of 5% and 20% for a FPR of 10%, *p* = .0076				
Fetal heart rate + free‐βhCG				
Fetal heart rate	0.0757	0.0277	1.0787 (1.0217, 1.1388)	.0062
Free‐βhCG	0.0077	0.0046	1.0077 (0.9987, 1.0168)	.0929
Intercept	−16,1894			
AUC = 0.6492, (95% CI = 0.5561, 0.7422), AIC = 290.8, DR = 19% for a FPR of 5% and 25% for a FPR of 10%, *p* = .0039				
Preterm birth < 34 weeks				
Fetal heart rate				
Fetal heart rate	0.0897	0.0445	1.0938 (1.0024, 1.1936)	.0439
Intercept	−19.2057			
AUC = 0.6750, (95% CI = 0.5388, 0.8112), AIC = 135.7, DR = 20% for a FPR of 5% and 25% for a FPR of 10%, *p* = .0367				
Fetal heart rate + free‐βhCG				
Fetal heart rate	0.0967	0.0471	1.1015 (1.0044, 1.2081)	.0401
Free‐βhCG	0.0118	0.0061	1.0119 (0.9999, 1.0241)	.0523
Intercept	−20.9344			
AUC = 0.7489, (95% CI = 0.6169, 0.8808), AIC = 124.1, DR = 18% for a FPR of 5% and 36% for a FPR of 10%, *p* = .0044				
Spontaneous preterm birth				
Spontaneous preterm birth <37 weeks				
Fetal heart rate				
Fetal heart rate	0.0889	0.0324	1.0929 (1.0256, 1.1647)	.0062
Intercept	−18,4048			
AUC = 0.6467, (95% CI = 0.5395, 0.7539), AIC = 226.5, DR = 15% for a FPR of 5% and 21% for a FPR of 10%, *p* = .0158				
Fetal heart rate + free‐βhCG				
Fetal heart rate	0.0895	0.0334	1.0936 (1.0243, 1.1677)	.0074
Free‐βhCG	0.0091	0.0051	1.0092 (0.9991, 1.0193)	.0735
Intercept	−18.9001			
AUC = 0.6800, (95% CI = 0.5716, 0.7885), AIC = 215.4, DR = 18% for a FPR of 5% and 27% for a FPR of 10%, *p* = .0038				
Fetal heart rate + hypothyroidism				
Fetal heart rate	0.0879	0.0325	1.0919 (1.0246, 1.1638)	.0068
Hypothyroidism	0.8717	0.4625	2.3909 (0.9657, 5.9195)	.0595
Intercept	−18.4540			
AUC = 0.6733, (95% CI = 0.5663, 0.02, AIC = 225.4, DR = 26% for a FPR of 5% and 26% for a FPR of 10%, *p* = .0044				
Fetal heart rate + free‐βhCG + hypothyroidism^*^				
Fetal heart rate	0.0879	0.0334	1.0919 (1.0228, 1.1659)	.0084
Free‐βhCG	0.0093	0.0051	1.0093 (0.9992, 1.0195)	.0699
Hypothyroidism	0.7238	0.4906	2.0623 (0.7884, 5.3948)	.1401
Intercept	−18.8107			
AUC = 0.6923, (95% CI = 0.5835, 0.8010), AIC = 215.5, DR = 27% for a FPR of 5% and 32% for a FPR of 10%, *p* = .0020				
Spontaneous preterm birth < 34 weeks				
Fetal heart rate				
Fetal heart rate	0.1121	0.0548	1.1187 (1.0047, 1.2455)	.0407
Intercept	−23.3101			
AUC = 0.7298, (95% CI = 0.6139, 0.8457), AIC = 96.77, DR = 20% for a FPR of 5% and 25% for a FPR of 10%, *p* = .0039				
Fetal heart rate + free‐βhCG				
Fetal heart rate	0.12964	0.05958	1.1384 (1.0129, 1.2794)	.0296
Free‐βhCG	0.01203	0.00781	1.0121 (0.9967, 1.0277)	.1233
Intercept	−26,84502			
AUC = 0.8111, (95% CI = 0.7198, 0.9024, AIC = 85.37, DR = 14% for a FPR of 5% and 43% for a FPR of 10%, *p* = .0045				
Fetal heart rate + free‐βhCG + hypothyroidism				
Fetal heart rate	0.1266	0.0593	1.1349 (1.0104, 1.2748)	.0328
Free‐βhCG	0.0119	0.0078	1.0119 (0.9966, 1.0276)	.1274
Hypothyroidism	0.7381	0.8502	2.0919 (0.3952, 11.0733)	.3853
Intercept	−26.4933			
AUC = 0.7891, (95% CI = 0.6752, 0.9030, AIC = 86.71, DR = 29% for a FPR of 5% and 29% for a FPR of 10%, *p* = .0083				

AIC, Akaike information criteria; AUC, area under the curve; free β‐hCG, free beta human chorionic gonadotropin; OR, odds ratio; PAPP‐A: pregnancy associated plasma protein‐A; *Pregestational or gestational hypothyroidism.

The highest predictive performance of FHR alone was observed for sPTB <34 weeks (AUC 0.73; detection rate 25% at a 10% false‐positive rate). For PTB <34 weeks and sPTB before 37 weeks, model performance was more modest (AUCs 0.65–0.68).

The combination of FHR and free‐βhCG improved predictive performance, particularly for sPTB <34 weeks (AUC 0.81; detection rate 43% at a 10% false‐positive rate). Adding maternal thyroid status to the models did not result in further improvement.

### FHR Cut‐off Analysis

The diagnostic performance of different FHR cut‐offs is shown in Table 5. The most favorable balance between sensitivity and specificity was observed for sPTB <34 weeks using a cut‐off of 170 bpm, yielding a detection rate of 25%, a false‐positive rate of 6%, a positive predictive value of 6.3%, and a negative predictive value of 97%.

**Table 5:** Behavior of the Fetal Heart Rate as a Marker of Preterm Birth and Spontaneous Preterm Birth in Our Pregnancy Population According to 3 Different Cut‐Offs

Cut‐off	PTB (N)	AUC	S (DR) (%)	Sp (%)	FPR (%)	FNR (%)	PPV (%)	NPV (%)
Any preterm birth								
<37 weeks								
>170 bpm	34	0.63	18	93	7	82	6	98
>167 bpm	34	0.63	23	85	15	77	4	98
>165 bpm	34	0.63	32	78	22	68	4	78
<34 weeks								
>170 bpm	12	0.67	25	94	6	75	4	94
>167 bpm	12	0.67	25	85	15	75	2	99
>165 bpm	12	0.67	25	78	22	75	1	99
Spontaneous preterm birth								
<37 weeks								
>170 bpm	23	0.65	17	94	6	82	5	98
>167 bpm	23	0.65	26	85	15	74	3	98
>165 bpm	23	0.65	35	78	22	65	3	98
<34 weeks								
>170 bpm	8	0.73	25	94	6	75	2	99
>167 bpm	8	0.73	25	85	15	75	1	99
>165 bpm	8	0.73	25	72	28	75	1	99

In the interest of clarity, percentages were rounded to the nearest unit, and AUCs were expressed with 2 decimals. AUC, area under the curve; S, (sensitivity); DR, detection rate; Sp, specificity; FPR, false positive rate (1‐specificity); FNR, false negative rate (1‐sensitivity); PPV, positive predictive value; NPV, negative predictive value.

## Discussion

### Principal Findings

This prospective validation study demonstrates that elevated FHR at the 12‐week scan is associated with an increased risk of PTB, particularly sPTB before 34 weeks' gestation. Among all clinical, sonographic, and biochemical parameters assessed during first‐trimester screening, FHR emerged as the only variable consistently associated with all PTB outcomes. Moreover, combining FHR with free‐βhCG substantially improved predictive performance for early sPTB.

### Comparison with Existing Literature

Efforts to identify first‐trimester predictors of sPTB have focused on maternal characteristics, biochemical markers, and early sonographic parameters (Table 6). Models based on maternal demographic and obstetric history alone have generally shown limited predictive ability, with moderate performance only for early sPTB outcomes.^13^, ^14^, ^15^, ^16^, ^17^, ^18^

**Table 6:** Review of Recently Studied Individual Markers for the Prediction of Preterm Birth and Spontaneous Preterm Birth Before 37 and 34 Weeks

Author and Reference	Parameter Investigated	Year	*N*	Outcome	% of PTB	GA Exam	AUC
Maternal factors							
Beta J et al^13^	Maternal factors	2011	33070	sPTB <34	353 (1.1)	11–14	0.67
Beta J et al^13^	Maternal factors (nulliparous women)	2011	33070	sPTB <34	353 (1.1)	11–14	0.61
Beta J et al^13^	Maternal factors (multiparous women)	2011	33070	sPTB <34	353 (1.1)	11–14	0.71
Greco E et al^14^	Maternal factors	2012	9974	sPTB <34	104 (1)	11–13	0.71
Greco E et al^14^	Maternal factors	2012	9974	sPTB 34–37	213 (2.1)	11–13	0.56
Feng Q et al^15^	Maternal factors	2025	3658	sPTB <34	19 (0.5)	11–14	0.70
Feng Q et al^15^	Maternal factors	2025	3658	sPTB <37	154 (4.2)	11–14	0.58
Becking EC et al^16^	Maternal factors	2025	56110	sPTB <37	1891 (3.4)	11–14	0.63
Goetzinger et al^17^	Maternal factors	2012	578	PTB <37	78 (13.5)	11–14	0.69
Goetzinger et al^17^	Maternal factors	2012	578	PTB <34	36 (6.2)	11–14	0.73
Goetzinger et al^17^	Maternal factors	2012	578	sPTB <34	36 (6.2)	11–14	0.78
Becerra Mojica CH et al^18^	History of PTB	2024	667	sPTB <34	12 (1.8)	11–14	0.58
Becerra Mojica CH et al^18^	History of PTB	2024	667	sPTB <37	61 (9.2)	11–14	0.57
Biochemical markers							
Goetzinger et al^17^	ADAM12	2012	578	PTB <37	78 (13.5)	11–14	0.60
Goetzinger et al^17^	PAPP‐A	2012	578	PTB <37	78 (13.5)	11–14	0.59
Goetzinger et al^17^	ADAM12	2012	578	PTB <34	36 (6.2)	11–14	0.63
Goetzinger et al^17^	PAPP‐A	2012	578	PTB <34	36 (6.2)	11–14	0.62
Weiner CP et al^19^	Plasma RNA apolipoprotein A1 (APOA1)	2023	60	sPTB <32	40 (66)	12–14	0.72
Weiner CP et al^19^	Plasma RNA proteasome activator subunit 2 (PSME2)	2023	60	sPTB <32	40 (66)	12–14	0.65
Mavreli et al^20^	miRNA‐125	2022	68	sPTB 32–37	34 (50)	11–13	0.89
Mavreli et al^21^	Vascular cell adhesion molecule 1 (VCAM1)	2023	68	sPTB 32–37	34 (50)	11–13	0.82
Mavreli et al^21^	Serum amyloid A‐1 protein (SAA1)	2023	68	sPTB 32–37	34 (50)	11–13	0.96
Mavreli et al^21^	Cytoskeletal protein Talin‐1	2023	68	sPTB 32–37	34 (50)	11–13	0.89
Becking EC et al^16^	Fetal fraction	2025	56110	PTB <37	1891 (3.4)	11–14	0.63
Becerra‐Mojica CH et al^23^	Complement factor H	2024	355	PTB <37	27 (7.6)	11–14	NR
Broekhuis A et al^24^	Ferritin	2024	2044	PTB <37	100 (4.9)	12	NR
Swierzc G et al^25^	PAPP‐A	2024	1164	PTB <37	84 (7.2)	11–14	0.64
Swierzc G et al^25^	PAPP‐A	2024	1164	sPTB <37	64 (5.5)	11–14	0.60
Swierzc G et al^25^	Free‐β‐hCG	2024	1164	PTB <37	84 (7.2)	11–14	0.59
Swierzc G et al^25^	Free‐β‐hCG	2024	1164	sPTB <37	64 (5.5)	11–14	0.61
Yildiz E et al^26^	Myosin‐binding protein C (MyBP‐C)	2023	628	PTB <37	45 (7.2)	11–14	0.73
Sonographical markers							
Greco E et al^14^	Cervical length	2012	9974	sPTB <34	104 (1)	11–13	0.78
Greco E et al^14^	Cervical length	2012	9974	sPTB (34–37)	213 (2.1)	11–13	0.55
Feng Q et al^15^	Cervical angle E	2025	3658	sPTB <34	19 (0.5)	11–14	0.74
Feng Q et al^15^	Cervical angle D1	2025	3658	sPTB <37	154 (4.2)	11–14	0.57
Feng Q et al^15^	Cervical angle D2	2025	3658	sPTB <37	154 (4.2)	11–14	0.57
Goetzinger et al^17^	Uterine artery Doppler	2012	578	PTB <37	78 (13.5)	11–14	0.54
Becerra Mojica CH et al^18^	Cervical consistency index <10^th^ centile	2024	667	sPTB <37	61 (9.2)	11–14	0.55
Becerra Mojica CH et al^18^	Cervical consistency index <10^th^ centile	2024	667	sPTB <34	12 (1.2)	11–14	0.62
Becerra Mojica CH et al^18^	Cervical length	2024	667	sPTB <37	61 (9.2)	11–14	0.50
Becerra Mojica CH et al^18^	Cervical length	2024	667	sPTB <34	12 (1.8)	11–14	0.41

PTB, preterm birth; PTB <34, PTB before 34 weeks; sPTB, spontaneous PTB; sPTB <34, spontaneous PTB <34 weeks; PAPP‐A, pregnancy‐associated plasma protein‐A; free‐βhCG, free beta human chorionic gonadotropin; NR, no reported; %PTB, percentage of PTB in the study; GA Exam, gestational age at exam; AUC, area under the curve.

Several studies have investigated first‐trimester biochemical markers associated with subsequent PTB, including circulating RNA, microRNAs, inflammatory proteins, and placental‐derived molecules.^19^, ^20^, ^21^, ^22^, ^23^, ^24^, ^25^ While some markers have shown promising predictive accuracy, most require specialized laboratory techniques and are not yet suitable for routine clinical use.

First‐trimester sonographic markers have also been explored, particularly cervical length and cervical morphology and consistency.^14^, ^15^, ^18^, ^26^ However, these measurements are technically challenging early in pregnancy, demonstrate variable reproducibility, and generally yield modest predictive performance, limiting their widespread implementation, especially in low‐resource settings.

In contrast, FHR is routinely measured during first‐trimester ultrasound, requires no additional training or equipment, and is highly reproducible. In the present study, FHR alone showed moderate predictive accuracy for sPTB <34 weeks, which increased substantially when combined with free‐βhCG. This combination aligns with the concept of leveraging routinely collected first‐trimester data to enhance early risk stratification.

### Possible Biological Mechanisms

The pathophysiological mechanisms linking elevated FHR at the 12‐week scan with subsequent sPTB are likely multifactorial and remain incompletely understood. One plausible pathway involves early exposure to maternal or placental inflammatory mediators, including cytokines and chemokines, which may influence fetal autonomic regulation and increase heart rate.^27^ Subclinical intra‐amniotic infection or mild inflammatory activation has been associated with abnormal FHR patterns, and such early inflammatory stimuli could initiate a cascade leading to premature activation of labor pathways.^28^, ^29^, ^30^, ^31^

Biochemical markers, such as elevated free β‐human chorionic gonadotropin (free‐βhCG), may reflect underlying placental dysfunction or early stress responses, which could amplify fetal autonomic perturbations. Together, elevated FHR and abnormal free‐βhCG levels may indicate a fetal‐placental environment under stress, integrating inflammatory, endocrine, and metabolic signals that increase the risk of early spontaneous labor.

In addition, maternal endocrine or metabolic alterations, including hypothyroidism, pregestational diabetes,^27^ or oxidative stress, could modulate fetal autonomic tone and contribute to subtle dysregulation of the sympathetic‐parasympathetic balance. FHR itself is a marker of early fetal autonomic maturation, and elevated rates may reflect fetal adaptation to intrauterine stressors, which could predispose to preterm labor.

Although we assessed indirect markers of inflammation, such as leukocyte counts, more sensitive biomarkers (eg, interleukin‐6, TNF‐α) were not available in this cohort and warrant further investigation.^32^ Understanding the interplay between early FHR elevation, placental signals such as free‐βhCG, and maternal‐fetal stress responses may help clarify the biological basis of early sPTB and identify additional predictive markers for integrated first‐trimester screening strategies.

### Clinical Implications

In clinical practice, prediction is rarely based on a single parameter, and screening strategies usually combine markers obtained at different stages of pregnancy. In this context, FHR should not be considered an independent trigger for intervention but rather an early marker of increased risk that may contribute to first‐trimester risk stratification. Moreover, elevated FHR could help identify a subgroup of pregnancies that might benefit from targeted second‐trimester surveillance, particularly cervical length measurement at around 20 weeks' gestation. Such a 2‐step screening strategy, combining first‐trimester FHR with second‐trimester cervical assessment, could improve the early identification of pregnancies at risk while avoiding unnecessary interventions.

However, given the modest detection rate and the limited number of cases in our cohort, FHR alone should not currently guide preventive treatment decisions. Further studies are needed to confirm these findings and to determine the potential role of FHR in combined screening models for sPTB.

If confirmed in larger populations, incorporating FHR into first‐trimester screening algorithms could enable earlier identification of women at increased risk of sPTB, allowing timely preventive interventions or closer surveillance. Importantly, FHR measurement is simple, inexpensive, and universally available during routine first‐trimester ultrasound, making it a particularly attractive marker for widespread clinical implementation, including in low‐resource settings.

This approach may further support the concept of the “inverted pyramid” of prenatal care proposed by Nicolaides^33^ by enabling simultaneous first‐trimester screening for chromosomal abnormalities, early preeclampsia, and sPTB using parameters that are already routinely collected.

### Strengths and Limitations

Key strengths of this study include its prospective design, strict definition of sPTB, validation in an independent regional population, and robust statistical methodology. Limitations include the relatively small number of PTB events, the absence of additional biochemical or inflammatory markers that could enhance predictive accuracy, and the inability to definitively elucidate the underlying biological mechanism linking elevated FHR to sPTB.

## Conclusion

Elevated FHR at the 12‐week scan is independently associated with sPTB, particularly before 34 weeks' gestation. Given its simplicity, reproducibility, and universal availability during routine first‐trimester ultrasound, FHR represents a promising early marker that might complement biochemical parameters such as free‐βhCG in future multimodal screening strategies for sPTB.

## Data Availability

The data that support the findings of this study are available from the corresponding author upon reasonable request.
